# No Difference in Short-Term Complications following Treatment of Closed Tibial Shaft Fractures with Intramedullary Nailing versus Plate Fixation

**DOI:** 10.1155/2023/1627225

**Published:** 2023-10-12

**Authors:** Conor N. O'Neill, Nicholas Hooper, Jacob Wait, James Satalich, David Cinats, Clarence Toney, Paul Perdue, Jibanananda Satpathy

**Affiliations:** Virginia Commonwealth University Health System, Richmond, VA, USA

## Abstract

**Objectives:**

Tibial shaft fractures are treated with both intramedullary nailing (IMN) and plate fixation (ORIF). Using a large national database, we aimed to explore the differences in thirty-day complication rates between IMN and ORIF.

**Methods:**

Patients in the American College of Surgeons National Surgical Quality Improvement Program (ACS NSQIP) database who had undergone either tibial IMN or ORIF for closed fractures from 2010 to 2018 were identified using current procedural terminology (CPT) codes. After excluding all patients with open fractures, the propensity score was matching. Univariate and multivariate logistic regressions were used to identify risk factors associated with the thirty-day incidence of complications in the two cohorts.

**Results:**

A total of 5,400 patients were identified with 3,902 (72.3%) undergoing IMN and 1,498 (27.7%) ORIF. After excluding any ICD-10 diagnosis codes not pertaining to closed, traumatic tibial shaft fractures, 2,136 IMN and 621 ORIF cases remained. After matching, the baseline demographics were not significantly different between the cohorts. Following matching, the rate of any adverse event (aae) did not differ significantly between the IMN (7.08% (*n* = 44)) and ORIF (8.86% (*n* = 55)) cohorts (*p*=0.13). There was also no significant difference in operative time (IMN = 98.5 min, ORIF = 100 min; *p*=0.3) or length of stay (IMN = 3.7 days, ORIF = 3.3 days; *p*=0.08) between the cohorts.

**Conclusion:**

There were no significant differences in short-term complications between cohorts. These are important data for the surgeon when considering surgical management of closed tibial shaft fractures.

## 1. Introduction

Tibial shaft fractures are the most treated long bone fracture with both intramedullary nailing (IMN) and plate fixation (ORIF) being reasonable options for management; however, IMN is used more frequently in treatment of these fractures [1]. In addition to IMN and ORIF, closed tibial shaft fractures can also be managed nonoperatively if certain criteria are met on imaging and physical exam. Tibial shaft fractures are more common in males, have a bimodal distribution peaking at ages 21 and 47, and frequently occur by high-energy mechanisms [2, 3]. These fractures often present with associated injuries, including other musculoskeletal and internal organ injuries which can influence both treatment modality and timeline [2].

IMN and ORIF are both acceptable treatment options in closed tibial shaft fractures, but some recent studies have demonstrated a difference in the complication rate following these procedures. In a meta-analysis by Lin et al., they found decreased wound complications with IMN with no significant differences in delayed union or nonunion between ORIF and IMN in extra-articular distal tibial fractures [4]. In another meta-analysis examining the effects of IMN versus minimally invasive percutaneous plating (MIPP), researchers found that MIPP resulted in shorter fracture healing time and lower rates of postoperative delayed union and pain when compared to IMN in tibial shaft fractures [5].

Several randomized control trials (RCT) have examined the differences in short term outcomes following ORIF or IMN, and there have been demonstrated benefits of both treatment options. Mukherjee and colleagues found no difference in the average time to union between ORIF and IMN; however, ORIF had an increased rate of complications, decreased incidence of persistent pain or chronic symptoms, and a better lower extremity function score (LEFS) compared to IMN in open or closed diaphyseal or metadiaphyseal fractures [6]. In contrast, another study found that there was increased malalignment in IMN versus ORIF, but no difference in infection rate, nonunion, and secondary procedures for extra articular open and closed tibial shaft fractures [7]. In addition, Saied and colleagues found that patients treated with IMN were more likely to need additional surgeries to achieve union and were also more likely to have pain in their limbs or knees compared to ORIF in patients with open and closed diaphyseal tibial fractures [8].

Recent studies show variable benefits and complications between IMN and ORIF, but these studies are limited by their size and design. To date, there has been no large-scale matched retrospective database study examining short-term complication rates in closed tibial shaft fractures for IMN versus ORIF.

The purpose of this study is to primarily compare 30-day complication rates between IMN and ORIF using the American College of Surgeons National Surgical Quality Improvement Program (ACS NSQIP) database. In addition, we seek to investigate more recent data to identify risk factors for complications in patients undergoing IMN and ORIF. By examining these data, surgeons will have more information which will aid in the decision-making process when choosing between IMN and ORIF for a patient.

## 2. Methods

### 2.1. Study Population

This is a retrospective cohort study of prospectively collected data as part of the ACS-NSQIP.

### 2.2. Data Collection

The ACS-NSQIP registry contains demographics, comorbidities, and laboratory values with corresponding readmission and complication rates within thirty days of the indexed procedure. Patients are identified through current procedural terminology (CPT) and International Classification of Diseases Ninth and Tenth Revision (ICD-9, ICD-10) codes [9]. NSQIP hospitals each employ trained nurse surgical clinical reviewers (SCRs) to oversee data collection adding an additional quality measure. All patients are monitored for thirty days postoperatively for any adverse events, readmissions, and reoperations. No outcome differences exist between institutions participating in the NSQIP program with nonparticipants (Molina 2015). The ACS-NSQIP database is comprised of a network of hospitals which are required to employ SCRs to collect 274 variables from surgical procedures. The database implements several quality assurance measures, such as biweekly random internal audits, which have reported less than 1.8% inter-rater disagreement [10, 11].

Due to the limitations with the NSQIP database, only patients with closed diaphyseal fractures were included in this review. Patient characteristics along with mechanism of injury were not able to be collected due to database restrictions along with mechanism.

Patients who had undergone an intermedullary nailing or ORIF for closed, diaphyseal fractures based on primary current procedural terminology (CPT) codes were identified from the ACS NSQIP database from January 1, 2010, through December 31, 2018. Patient demographics, including age, smoking status, body mass index (BMI), gender, and American Society of Anesthesiologists' (ASA) physical status classification score was collected along with complications data for each. After excluding all patients with open or nontraumatic fractures (i.e., pathologic), the nearest neighbor propensity score matching was used to address any potential demographic differences between the IMN and ORIF cohorts. Patients in each group then underwent a 1 : 1 propensity match for age, gender, BMI, and ASA status, diabetes mellitus (DM), hypertension requiring medication, congestive heart failure (CHF), chronic obstructive pulmonary disorder (COPD), and bleeding disorders. Unfortunately, we were unable to include patient smoking status in our matching model as it significantly limited the number of patients included in each cohort. However, after performing backward selection to control for smoking, we found that it did not have a significant effect on our model and was therefore not included in the final model.

For each patient, we recorded the LOS, specific 30-day complications, and readmission rate. Complications that were queried included rate of both superficial and deep surgical site infections (SSI), pneumonia, postoperative re-intubation or failure to wean from ventilator, pulmonary embolism (PE), postoperative renal insufficiency or failure, urine infection, stroke, cardiac arrest, myocardial infarction, transfusions, deep vein thrombosis (DVT), sepsis, and shock. We also recorded deaths and patients who required return to the operating room. Length of stay was defined as the number of days from procedure to postoperative discharge. Minor complications were superficial SSI, urinary tract infection (UTI), pneumonia, progressive renal insufficiency, or wound dehiscence. Severe complications included death, coma, placement on ventilator, unplanned intubation, stroke/cerebrovascular accident, thromboembolic event (DVT or PE), cardiac arrest, myocardial infarction (MI), acute renal failure, sepsis, septic shock, or return to the operating room.

### 2.3. Statistical Analysis

Statistical analysis was performed using R-studio software version 1.0.143 (R Foundation for Statistical Computing, Vienna, Austria). A number of statistical methods were employed for the data analysis, including propensity score matching, bivariate, and multivariable logistic regression. Patient demographics, comorbidities, and complications were compared between the two cohorts using the Student two-tailed *t*-test for continuous variables and chi-square analysis for categorical variables. The variables that showed significance from the univariate comparisons were used as the variables included in each of the multivariable logistic regression. Propensity score matching was carried out using the nearest neighbor method in order to reduce treatment assignment bias and ideally simulate randomization between the IMN and ORIF cohorts. Multivariable logistic regression with robust error variance was used to identify risk factors for complications after IMN and ORIF. Throughout these analyses, statistical significance was achieved with *p* < 0.05.

## 3. Results

### 3.1. Cohort Characteristics

**Figure 1:**
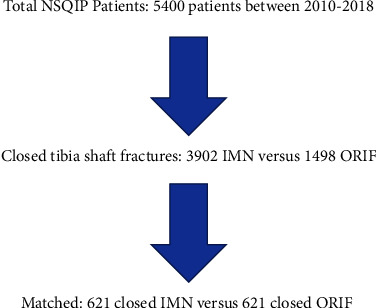
Flow diagram of data.

A total of 5,400 patients were identified with 3,902 (72.3%) undergoing IMN and 1,498 (27.7%) ORIF between 2010 and 2018 (Table 1). After excluding any ICD-10 diagnosis codes not pertaining to closed, traumatic tibial shaft fractures, 2,136 IMN and 621 ORIF cases remained. Of these, 1,242 patients (621 from each of the inpatient and outpatient cohorts) were matched and included in the analysis (Figure 1). Matching was based on different age, sex, BMI, ASA class, smoking status, and diagnosis of diabetes. After propensity score matching, the cohorts did not demonstrate any significant differences with respect to any baseline demographic preoperative variables.

After matching, the IMN cohort (*N* = 621) had a mean age of 37.1 ± 19.6 years, with 45.7% (*n* = 284) male patients and a mean body mass index (BMI) of 28.9 ± 7.3 kg/m^2^, whereas for the ORIF cohort (*N* = 621) had a mean age of 36.8 ± 18.4 years, with 43.8% (*n* = 272) male and a mean BMI of 29.2 ± 7.6 kg/m^2^.

### 3.2. Postoperative Outcomes

Prior to matching, most recorded complications were in the ORIF group. Patients undergoing ORIF had significantly higher rates of wound dehiscence (*p*=0.040) and surgical site infection (SSI) (*p*=0.001). Although not statistically significant, there was an overall higher rate of any adverse event in the ORIF group (8.86%) versus the IMN group (6.78%) (*p*=0.088) (Table 2). Following matching, the rate of any adverse event (aae: surgical site infection, renal insufficiency, intubation, pneumonia, deep vein thrombosis (DVT), pulmonary embolism, urinary tract infection) did not differ significantly between the IMN (7.09% (*n* = 44)) and ORIF (8.86% (*n* = 55)) cohorts (*p*=0.292). There was also no significant difference in operative time (IMN = 98.5 min, ORIF = 100 min; *p*=0.3) or length of stay (IMN = 3.7 days, ORIF = 3.3 days; *p*=0.08) between the cohorts.

When accounting for all baseline demographics, each preoperative steroid use (odds ratio [OR]: 3.60, 95% confidence interval [CI]: 1.61–7.93; *p*=0.002) and preoperative transfusion (OR: 5.42, CI: 1,79−16.42, *p*=0.003) increased the risk for any adverse event. There was also a statistically significant increase in the risk of complication with increasing age (OR: 1.02, CI: 1.01–1.04, *p*=0.004) and operative time (OR: 1.004, CI: 1.003–1.005, *p*=0.003). In addition, insulin dependent diabetes mellitus (IDDM) was significantly associated with increase in the odds of developing adverse events during surgery (OR: 0.53, CI: 0.29–0.95, *p*=0.034). The presence of preoperative noninsulin dependent diabetes mellitus (NIDDM) was not associated with significantly increased odds of adverse events (OR: 0.66, CI: 0.29–1.53, *p*=0.37). Lastly, increasing ASA class also increased the odds of complication. There was no significant increase in the odds of adverse events for ASA class 2, while both ASA class 3 (OR: 13.367, CI: 1.70–105.12, *p*=0.014) and ASA class 4 (OR: 15.343, CI: 1.62–145.03, *p*=0.017) were significantly associated with increase odds of any adverse events.

## 4. Discussion

Closed tibial shaft fractures are common lower extremity injuries that frequently require surgical treatment. Although there are several treatment modalities, both operative and nonoperative, surgical stabilization typically involves intramedullary nailing or open reduction and internal fixation. Neither of these methods have been shown to be definitively superior to the other, and the decision of which surgical technique is used may be influenced by a combination of bony and soft tissue injury, concomitant injuries, medical comorbidities, and surgeon preference.

To date, there has been sparse and conflicting data comparing IMN and ORIF for closed tibial shaft fractures. Our results showed that there is no difference in short term adverse event rates when tibial shaft fractures are treated using IMN or ORIF after matching. The rate of any surgical adverse event for IMN and ORIF was 7.09% and 8.86%, respectively, and 30-day return to OR rates for IMN and ORIF was 1.61% and 2.25%, respectively. These results are similar to previously published rates of reoperation for these respective procedures [1, 12]. Of note, there was a higher rate of surgical site infection and wound dehiscence in the ORIF group; however, this effect disappeared after matching, indicating that it was most likely seen due to confounding. This suggests there could be a difference in the type of patients that surgeons choose to operate on using IMN or ORIF. In addition, there was no significant difference in the operative time or hospital length of stay between the two groups.

Our team also sought to identify risk factors for complications after IMN or ORIF. It was found that increasing age, receiving a transfusion, increased operative-time, steroid use, and ASA class 3 or 4 were independently associated with an increase in the odds of developing any adverse event during surgery (Table 3). A previous study by Minhas and colleagues only reported that there was an increased rate of postoperative transfusion requirements with IMN as compared to ORIF. However, we showed each of these factors to play a significant role in complication occurrence when taking into account all demographics, comorbidities, and operative procedures. Other studies have shown similar findings associated with all of these factors including an increased risk of postoperative complications with increasing age ([13–15]), increasing ASA class ([16, 17]), steroid use ([18, 19]), and receiving a transfusion. Despite these factors being previously identified as risk factors for surgical complications, their association with risk during treatment of closed tibial shaft fractures may help guide evaluation and management for these patients and should be given consideration in the development of risk stratification calculations.

As previously stated, the present study found that there is a significant effect of age and ASA class on the development of complications. Our study demonstrates that, with every increase in age of 1-year, the odds ratio of developing a complication is 1.023. Furthermore, our study showed that an ASA class of 3 or 4 increased the odds of complication development by 13.37 and 15.34, respectively (Table 3). The ASA classification is a commonly used tool for patients to be stratified based on their baseline physical status and provide a quick reference for anesthesiologists and surgical teams to understand the patient's overall preoperative health, but it is not meant to be used as a predictor of risk. However, in this study we show that ASA classification does act as a predictor of complication risk with those patients with ASA class 3 and 4 having a large significant odds ratio for the development of any adverse event following IMN or ORIF. Nonetheless, these risk factors, along with receiving a transfusion, increased operative time, and steroid use, may be useful to include in risk calculators, such as the NSQIP risk calculator. The inclusion of these baseline characteristics in risk calculators for individuals undergoing ORIF or IMN may better risk stratify patients preoperatively and therefore influence their management.

Understanding the risk factors which predispose a patient to having more postoperative complications will aid in screening in the time after surgery. Patients with these risk factors for complications may need more frequent and intensive follow-up visits to rule out and treat complications that could arise. In addition, understanding the risk factors which predispose a patient to increased complications may aid the surgeon in deciding between conservative and surgical management. A patient who fits the characteristics of a patient most likely to have complications after surgery may benefit from more conservative management, assuming that mandatory operative criteria for closed tibial shaft fractures are met, and there is no emergent surgical indication present.

There has been mixed evidence surrounding the complications following IMN and ORIF. In a meta-analysis comparing IMN versus ORIF for treatment of distal tibial fractures by Ekman and colleagues, it was found that IMN was associated with decreased rates of postoperative complications including a decreased rate of deep infection, delayed wound healing, superficial infection, and soft-tissue irritation. Similarly, our study found that there was an increase in surgical site infection and wound dehiscence with ORIF prior to matching, but this effect disappeared after matching. Given that the studies in the Ekman et al. meta-analysis were RCTs, it is unlikely that confounding played a role in their finding; however, many of the studies included had small sample sizes and were prone to the risk of systematic biases (Ekman). Further studies are needed to fully understand the infection and soft tissue complications following surgical treatment of closed tibial shaft fractures.

However, in a similar study to the present one, Minhas and colleagues found that there was also no increasing risk of 30-day postoperative complications between plate fixation and IMN [12]. This ACS-NSQIP database study concluded that both surgical methods resulted in similar short-term complications and can both safely be used to treat closed tibial shaft fractures [12]. Since the time of this paper, there have been changes in surgical technology and ideology. Our study examines similar outcomes but with a timeline of 2010–2018 as opposed to the study period of Minhas et al.'s study which examined these outcomes from 2006–2012. Overall, our results confirm their findings that there is no difference in short term outcomes in the treatment of closed tibial shaft fracture following ORIF or IMN. There is an additional study using NSQIP database with regards to outcomes following UKA vs TKA [20], which could be used in addition for NSQIP quality metrics.

One possible explanation for the varied results from previous studies is that tibial shaft fractures are extremely varied in their mechanism of injury, anatomic fracture location, and other associated injuries including both open and closed fractures. In light of this, many studies which examine IMN vs ORIF for tibial shaft fractures limit their discussion to an anatomic location or complication profile. This study, which examined short-term complications of closed tibial shaft fractures, is also limited for this reason. Another limitation of studies which examine the treatment of tibial shaft fractures is that it is very complicated to remove surgeon bias from any RCT that reports on subjective features of disease or complications as it is challenging to blind the investigator or participant in a surgical treatment. In this study, the use of a database containing data which were not collected for this project limit the opportunity for this bias.

It is important to recognize the findings of this study in the context of its inherent limitations. First, as a large database study, the NSQIP data records on only certain patient characteristics and certain complications which precludes us from being able to comment on patient reported outcome scores, functional outcomes, or radiographic analyses. In addition, it is important to note that our data are limited to 30-day post-surgical outcomes due to the window of ACS-NSQIP data collection. This window may fail to capture long-term complications and secondary procedures such as hardware prominence, malunion rate, and a common complication of plating. Previous studies have resulted in conflicting data on the complication and success rates of IMN versus ORIF for tibial shaft fractures. Although both IMN and ORIF are considered acceptable treatment options for closed tibial shaft fractures, each have been associated with different notable complications in previous studies [7]. IMN has been associated with increased knee pain ([6, 8, 21, 22]) and malalignment ([7, 21, 23–26]), while ORIF is associated with increased rates of infection ([4, 21, 25, 26]). Because this study was focused on short-term outcomes, any possible advantage of ORIF for preventing future knee pain would not be seen. Additionally, our focus on complications which are accessible through the ACS-NSQIP database does not allow us to evaluate for rates of malalignment and malunion. Lastly, the NSQIP database lacks information regarding mechanism of injury, location of tibial fracture or potential soft tissue swelling, which may have influenced the procedure performed.

This study is unique as there are limited studies with regards to publications in the literature. The team presents a unique large scale database review on short-term outcomes regarding closed tibial shaft fracture 3.

Another limitation to this paper is with regards to database studies; the team did not have the ability to find patient factors or fracture classification. There were only closed diaphyseal tibia shaft fractures collected in this study without the OTA classification associated.

## 5. Conclusion

These data provide surgeons with updated information which will allow them to better choose between ORIF and IMN in their patient. Although the long-term benefits and complications of treating tibial shaft fractures with either ORIF or IMN are not clear, this study shows that there is not a significant change in short-term risks which allows the surgeon to consider each case individually to decide whether the approach, surgical trauma, and recovery course of each treatment option is more beneficial for the patient. Our secondary finding of factors which increase postoperative complications can help identify patients who are more likely to experience complications resulting in increased screening and earlier rates of detection of these complications. Further studies are needed to better understand the risks and benefits of different treatment methods for tibial shaft fractures.

## Figures and Tables

**Table 1:** Demographic and comorbidity characteristics for patients undergoing tibial ORIF and IMN.

	IMN unadjusted (%)	ORIF (%)	*p* value	IMN matched (%)	ORIF (%)	*p* value
Patients, *N* (%)	2136 (77.5)	621 (22.5)		621 (50)	621 (50)	
Age (years, mean ± SD)	31.1 ± 18.5	36.8 ± 18.4	<0.001^*∗*^	37.1 ± 19.6	36.8 ± 18.4	0.766
BMI (kg/m^2^, mean ± SD)	27.8 ± 7.1	29.2 ± 7.6	<0.001^*∗*^	28.9 ± 7.3	29.2 ± 7.6	0.358
Male sex	1174 (55.0)	272 (43.8)	<0.001^*∗*^	284 (45.7)	272 (43.8)	0.530
Operative time (mins)	99.1 ± 51.7	100.0 ± 47.5	0.660	98.5 ± 55.1	100.0 ± 47.5	0.601
Length of stay	3.55 ± 4.04	3.32 ± 5.76	0.366	3.71 ± 3.66	3.32 ± 5.76	0.160
*ASA class*	2.18 ± 0.8	2.27 ± 0.78	0.016^*∗*^	2.23 ± 0.77	2.27 ± 0.78	0.381
1 (no disturbance)	426 (19.9)	103 (16.6)	—	113 (18.2)	103 (16.6)	—
2 (mild disturbance)	971 (45.5)	268 (43.2)	—	266 (42.8)	268 (43.2)	—
3 (severe disturbance)	652 (30.5)	228 (36.7)	—	225 (36.2)	228 (36.7)	—
4 (life-threatening disturbance)	87 (4.1)	21 (3.4)	—	17 (2.7)	21 (3.4)	—
5 (moribund)	0	1 (0.2)	—	0	1 (0.2)	—
*Race*						
White	1445 (67.6)	423 (68.9)	—	423 (68.9)	423 (68.9)	—
Black	199 (9.3)	57 (9.2)	—	57 (9.2)	57 (9.2)	—
Asian	69 (3.2)	16 (2.3)	—	16 (2.3)	16 (2.3)	—
Other	423 (19.8)	125 (20.1)	—	125 (20.1)	125 (20.1)	—
Dependent functional status (partial or total)	143 (6.7)	48 (7.7)	0.413	48 (7.7)	48 (7.7)	0.439
Current smoker	611 (28.6)	143 (23.0)	0.006∗	145 (23.3)	143 (23.0)	0.944
*Comorbidities, N (%)*						
Congestive heart failure	17 (0.80)	7 (1.1)	0.456	6 (0.97)	7 (1.1)	1
Renal failure	9 (0.42)	2 (0.32)	1	4 (0.64)	2 (0.32)	0.701
Dialysis	26 (1.2)	7 (1.1)	1	4 (0.64)	7 (1.1)	0.568
Steroid use	55 (2.6)	17 (2.7)	0.874	19 (3.1)	17 (2.7)	0.857
Weight loss	5 (0.23)	2 (0.32)	1	4 (0.64)	2 (0.32)	0.693
Bleeding disorder	107 (5.0)	29 (4.7)	0.761	35 (5.6)	29 (4.7)	0.526
Ascites	0	0	1	0	0	1
Preoperative transfusion	22 (1.0)	5 (0.81)	0.666	11 (1.8)	5 (0.81)	0.202
Diabetes	271 (12.7)	103 (16.6)	0.010^*∗*^	86 (13.8)	103 (16.6)	0.196
IDDM	153 (7.2)	54 (8.7)	—	52 (8.4)	54 (8.7)	—
NIDDM	118 (5.5)	49 (7.9)	—	34 (5.4)	49 (7.9)	—
DOE	60 (2.8)	26 (4.2)	0.895	17 (2.7)	26 (4.2)	0.226
COPD	90 (4.2)	24 (3.9)	0.787	25 (4.0)	24 (3.9)	1

BMI: body mass index; COPD: chronic obstructive pulmonary disease; DOE: dyspnea on exertion; renal failure: wherein renal function has been compromised within 24 hrs prior to surgery; dialysis: acute or chronic renal failure requiring dialysis within 2 weeks of indexed procedure; weight loss: considered as greater than 10% decrease in body weight in six-month interval preceding surgery; IDDM: insulin-dependent diabetes mellitus.

**Table 2:** Incidence of adverse events for patients undergoing tibial IMN vs ORIF.

	IMN (27759) unmatched		ORIF (27758) unmatched		*p*value	IMN matched		ORIF matched		*p*value	Overall matched	
	No. (2136)	Rate (%)	No.	Rate		No. (621)	Rate	No.	Rate		No. (1242)	Rate (%)
Any adverse event	145	6.78	55	8.86	0.088	44	7.09	55	8.86	0.292	99	7.97
Death	8	0.374	5	0.805	0.184	5	0.805	3	0.805	0.742	8	0.644
Wound dehiscence	1	0.046	3	0.483	0.040^*∗*^	0	0	3	0.483	0.264	3	0.242
Sepsis	10	0.468	3	0.483	1	2	0.322	3	0.483	1	5	0.403
Pulmonary embolism	7	0.328	2	0.322	1	2	0.322	2	0.322	1	4	0.322
Renal complication	6	0.281	0	0	0.342	2	0.322	0	0	0.493	2	0.161
Myocardial infarction	5	0.234	2	0.322	1	2	0.322	2	0.322	1	4	0.322
Cardiac arrest	2	0.094	0	0	1	0	0	0	0	1	0	0
Stroke	1	0.047	0	0	1	0	0	0	0	1	0	0
Transfusion	65	3.043	27	4.35	0.134	18	2.90	27	4.35	0.207	45	3.62
DVT	10	0.468	2	0.322	0.746	3	0.483	2	0.322	1	5	0.403
UTI	23	1.077	9	1.45	0.488	8	1.29	9	1.45	1	17	1.37
Pneumonia	37	1.732	8	1.29	0.492	10	1.61	8	1.29	0.821	18	1.45
Intubation issues	10	0.468	1	0.161	0.490	1	0.161	1	0.161	1	2	0.16
SSI	13	0.609	11	1.77	0.001^*∗*^	5	0.805	11	1.77	0.209	16	1.29
Return to the OR	28	1.311	14	2.25	0.093	10	1.61	14	2.25	0.528	24	1.93

Any adverse event (AAE): superficial and deep surgical site infection, organ space infection, renal failure or insufficiency, intubation (fail to wean or reintubation), post-operative transfusion, pneumonia, DVT, PE, UTI, stroke, cardiac arrest, MI–spell out, return to the OR; DVT: deep vein thrombosis; UTI: urinary tract infection; SSI: surgical site infection; OR: operating room; Intubation issues: re-intubation or failure to wean from intubation; Renal complication: progressive renal insufficiency or renal failure.

**Table 3:** Odds of developing any adverse event during surgery as related to patient demographics, comorbidities, and procedure.

	Multivariable analysis^*∗∗*^		
	OR coef.	95% CI	*p* value
*Overall*			
Age (1-year intervals)	1.023	1.007–1.040	0.004^*∗*^
Operative time (1 min intervals)	1.006	1.002–1.010	0.003^*∗*^

*ASA class*			
1	Ref	—	—
2	3.428	0.436–26.96	0.242
3	13.367	1.700–105.12	0.014^*∗*^
4	15.343	1.624–145.03	0.017^*∗*^

*Preop transfusion*			
No	Ref	—	—
Yes	5.416	1.786–16.422	0.003^*∗*^

*Steroid use*			
No	Ref	—	—
Yes	3.569	1.606–7.932	0.002^*∗*^

*Diabetes mellitus*			
IDDM	Ref	—	—
NIDDM	0.664	0.288–1.530	0.336
None	0.525	0.290–0.952	0.034^*∗*^

^*∗∗*^Variables are adjusted for all baseline characteristics. Coef: coefficient; 95% CI: 95% confidence interval; ref: reference; ASA: American society of anesthesiology; NIDDM: non-insulin dependent diabetes mellitus; IDDM: insulin-dependent diabetes mellitus.

## Data Availability

The data used to support the findings of this study are included within the article.
